# Design a New Strategy Based on Nanoparticle-Enhanced Chemiluminescence Sensor Array for Biothiols Discrimination

**DOI:** 10.1038/srep32160

**Published:** 2016-08-30

**Authors:** Maryam Shahrajabian, M. Reza Hormozi-Nezhad

**Affiliations:** 1 Department of Chemistry, Sharif University of Technology, Tehran, 11155-9516, Iran; 2 Institute for Nanoscience and Nanotechnology, Sharif University of Technology, Tehran, Iran

## Abstract

Array-based sensor is an interesting approach that suggests an alternative to expensive analytical methods. In this work, we introduce a novel, simple, and sensitive nanoparticle-based chemiluminescence (CL) sensor array for discrimination of biothiols (e.g., cysteine, glutathione and glutathione disulfide). The proposed CL sensor array is based on the CL efficiencies of four types of enhanced nanoparticle-based CL systems. The intensity of CL was altered to varying degrees upon interaction with biothiols, producing unique CL response patterns. These distinct CL response patterns were collected as “fingerprints” and were then identified through chemometric methods, including linear discriminant analysis (LDA) and hierarchical cluster analysis (HCA). The developed array was able to successfully differentiate between cysteine, glutathione and glutathione disulfide in a wide concentration range. Moreover, it was applied to distinguish among the above analytes in human plasma.

In recent years, the use of nanoparticles to catalyze chemiluminescence (CL) reactions has opened a new window to the application of CL as a powerful tool in analytical chemistry and has attracted many researchers. A variety of nanoparticles, such as metal, metal oxide, semiconductor and magnetic nanoparticles can exhibit the enhancement effect and have been widely employed in different CL systems. For example, silver, gold, and platinum nanoparticles were used to catalyze the chemiluminescent reaction including luminol-K_3_Fe(CN)_6_^1^, luminol-H_2_O_2_^2^, luminol-AgNO_3_^3^, lucigenin-KI^4^, KIO_4_-NaOH/Na_2_CO_3_^5^, lucigenin-hydrazine^6^, and lucigenin-ethanol^7^. The catalytic activity of Au-Ag alloy nanoparticles^8^, Co/Fe nanoparticles^9^, and Cu/Ni metal nanoparticles^10^ in liquid-phase CL reactions was also studied. It has been stated that the enhancement effect is due to increased surface area and surface electron density in chemiluminescence reactions containing nanoparticles.

It has also been reported that organic compounds containing hydroxyl (OH), amino (NH_2_), or mercapto (SH) groups can greatly interact with nanoparticles^11^. These compounds can interact with nanoparticles in CL reactions leading to enhancement or inhibition of the amplified CL signals, providing a suitable detection system. Accordingly, a number of prominent analytical methods have been reported. For example Cui *et al.* have reported that gold nanoparticles provide effective enhancement in a luminol-H_2_O_2_ CL system. Organic compounds and amino acides could effectively react with gold nanoparticles (AuNPs) to decrease the CL intensity. These compounds were quantified by use of a flow injection system^2^. In another study, the effect of eighteen amino acids and twenty-five organic compounds on the enhanced CL signal of a luminol–AgNO_3_– AgNPs system was studied, which led to an efficient detection of analytes^12^. Cui and co-workers have also investigated the influence of twenty amino acids on the the enhanced CL of luminol–H_2_O_2_–AgNPs^13^. A rhodamine 6G-cerium(IV)- Au-Ag alloy nanoparticles system was also studied, which led to an efficient detection of seventeen amino acids and twenty-two organic compounds^7^. Therefore, many investigations have demonstrated that the nanoparticle-assisted CL system is capable of detecting a wide variety of analytes.

Although considerable efforts have been dedicated to the detection of various analytes by means of this systems, the power of this approach in selectivity may be limited. Nowadays, the creation of selective CL detections is still a great challenge. Therefore, it is necessary to develop selective nanoparticles enhanced CL systems. As a clue to this problem, in the present contribution, we combined nanoparticle enhanced CL system with the sensor array technique.

Array-based sensors have become an interesting set of tools for analytical studies and their application continues to extend rapidly^14^. In recent years, many sensor array designs have been applied by various research groups for sensing and identifying different chemicals, such as biomacromolecules (Rotello *et al.*^15^ and Hamilton *et at.*^16^), vapors (Suslick *et al.*^17^, Lewis *et al.*^18^, Walt *et al.*^19^, and Rose *et al.*^20^), and ions (e.g., Anslyn *et al.*^21^, Anzenbacher *et al.*^22^, and Wolfbeis *et al.*^23^). Chemosensors are slowly shifting toward sensor arrays leaving behind expensive and non-versatile selective sensors. In chemical sensor array, also called chemical noses or chemical tongues, the specificity of the identification comes from the response pattern derived from the sensor elements. In sensor arrays, each non-specific sensor element has a special amount of selectivity to each analyte. By using a pattern recognition protocol, signals of all sensor elements are simultaneously analyzed and a fingerprint-like response pattern is generated for each analyte. These unique response patterns are used for the classification and identification of a set of analytes. A large number of chemical array sensors use changes in the optical properties of the sensing elements to provide distinct response patterns. For example, these optical properties may consist of changes in the absorption spectra (color) and the emission intensity. Recently a few number of chemiluminescent (CL) sensor arrays have been reported that are quite interesting. For instance, Cui *et al.* reported a new nanoparticle-based chemiluminescent sensor array for protein discrimination. In their work CL intensity, time for CL emissions to appear, and the time to reach the CL peak value were used as the sensing elements. Their system provided distinct response patterns which were discriminated using classical principal component analysis (PCA)^24^. A similar system was used for pesticide discrimination by He *et al.*^25^. Moreover Zhang *et al.* designed a sensor array based on nanomaterial-assisted thermochemiluminescence (TCL) for protein sensing and cell discrimination. They reported that the TCL of their analytes was generated on the surface of catalytic nanomaterials. Therefore, six catalytic nanomaterials, γ-Al_2_O_3_, Pt/Ba/Al, MgO, MgCO_3_, SrCO_3_, and ZrO_2_, were used as sensing elements. Unique TCL response patterns were obtained from the array and were analyzed by linear discriminant analysis (LDA)^26^.

In this study, three common biothiols for which we have recently introduced a colorimetric sensor array^27^, were chosen as model analytes in order to discriminate among them. The biothiols of interest included glutathione (GSH), glutathione disulfide (GSSG), and cysteine (Cys). These compounds have fundamental roles in biological processes, such as reversible redox reactions and enormous cellular functions such as metabolism and detoxification. Any change in the biothiols redox status, particularly for small molecular biothiols such as cysteine, glutathione disulfide, and glutathione can influence the physiological properties^28^.

Over the past decades, various analytical methods have been developed for the detection of biothiols, for instance, high performance liquid chromatography (HPLC)^29^, capillary electrophoresis^30^,^31^, mass spectrometry^32^, fluorometry^33^,^34^ and colorimetry^35^. Though all these methods show good capability for the detection of different thiols, they suffer from a variety of disadvantages, such as the use of sophisticated, expensive instruments, time-consuming and preliminary treatment. Several enzymatic methods have also been applied to determine biothiols^36^,^37^. Although these methods have good selectivity, the chemical stability of the enzymes is poor, which greatly restricts their simple use. Due to various advantages such as simple instrumentation, ease of control, rapidity in signal detection, and simple operation, we chose chemiluminescence (CL)-based assays for our study. In addition in comparison with reported array based systems 27,38 we achieved biothiols discrimination in a lower and wider concentration range. Hence, we developed an approach, which holds the promise of achieving both simplicity and sensitivity.

Different nanomaterials with different CL reagents were chosen, for the first time, as sensing elements. We used luminol-AgNO_3_ enhanced chemiluminescence (CL) in the presence of NaBH_4_ coated AuNPs (BH_4_-AuNPs) and citrate-capped AuNPs (Cit-AuNPs) in alkaline solution and luminol-H_2_O_2_ enhanced chemiluminescence (CL) in the presence of citrate coated AgNPs (Cit-AgNPs) and thioglycolic acid (TGA) functionalized cadmium telluride (CdTe) quantum dots (TGA-CdTe QDs) in alkaline solution as sensing elements. Combining nanomaterial-assisted CL probes with the sensor array design led to a selective and powerful approach for the discrimination of important biothiols. The developed CL sensor array produced distinct response patterns for the analytes of interest in a wide concentration range. Figure 1 shows a schematic diagram of the experimental setup. For discrimination purposes, chemometric methods, including linear discriminant analysis (LDA), and hierarchical cluster analysis (HCA), were employed.

## Result and Discussion

### Enhancement effect of nanoparticles on luminol CL

In a preliminary study, the enhancement effect of different nanoparticles on the luminol–H_2_O_2_ and luminol–AgNO_3_ CL was investigated within a flow injection system. As shown in Fig. 2, the results indicated that the CL signal of the luminol–H_2_O_2_ system was significantly enhanced by AgNPs and CdTe QDs, while the CL intensity of luminol–AgNO_3_ reaction was significantly enhanced by BH_4_-AuNPs and Cit-AuNPs. Therefore, we used these four nanoparticle-enhanced CL systems as sensor elements to generate response patterns. It is well-known and has been widely studied, that metal ions can catalyze the decomposition of H_2_O_2_ and therefore catalyze luminol–H_2_O_2_ CL systems. AgNPs are also able to efficiently catalyze the H_2_O_2_ decomposition. As shown in Fig. S1 H_2_O_2_ was decomposed by AgNPs to ^•^OH radicals, which further reacted with H_2_O_2_ to generate superoxide anions (O_2_^•−^) and luminol radicals. Furthermore, the mechanism of luminol–H_2_O_2_ CL system was reported due to the reaction of luminol radical with superoxide anion^2^,^13^. Silver nitrate is capable of oxidizing luminol but it doesn’t produce a strong CL signal under general conditions. In the presence of gold nanoparticles, luminol could react with AgNO_3_ and produce strong CL signal. As shown in Fig. S2, AgNO_3_ was reduced to Ag atoms and luminol was oxidized to luminol radical which were catalyzed by gold nanoparticles and happened on the surface of gold nanoparticles. Luminol radical was then followed by reaction with the dissolved oxygen, giving rise to light emission^12^,^39^,^40^,^41^. The possible mechanism of the enhanced luminol–H_2_O_2_ CL reaction induced by CdTe QDs was shown in Fig. S3 In the present system, we suppose that the H_2_O_2_ oxidant can oxidize CdTe QDs, producing cationic radicals R^+•^, or oxidized species (CdTe QDs)^+•^, which can act as enhancers for the luminol–H_2_O_2_ CL system to generate strong CL radiation^42^,^43^.

### Optimization of experimental conditions

The experimental conditions were optimized for the luminol–H_2_O_2_– Cit-AgNPs and luminol–AgNO_3_– Cit-AuNPs CL systems. Considering the CL intensity, standard deviation, and the amount of reagent consumption, the optimal conditions for CL systems were utilized for further experiments. As shown in Figs S4 and S5, the maximum CL signals were obtained in 0.4 mM luminol, 0.15 M H_2_O_2_, 20 rpm flow rate, 0.01 M NaOH for luminol–H_2_O_2_–AgNPs CL system and in. 0.5 mM luminol, 25 μM AgNO_3_, 25 rpm flow rate, and 0.1 M NaOH for luminol–AgNO_3_– Cit-AuNPs CL system.

### Inhibition and enhancement effects of biothiols on nanoparticle-based chemiluminescence

Organic compounds containing amino (NH_2_), hydroxyl (OH), and mercapto (SH) groups have been found to interact with nanoparticles^2^. The results demonstrate that such compounds interact with nanoparticles in the CL reaction leading to a change in CL intensity. In the present work, the effects of these biomolecules on the luminol–AgNO_3_–AuNPs and the luminol–H_2_O_2_–(AgNPs/CdTe QDs) CL systems has been investigated. Biothiols in wide concentration ranges were exposed to different types of NPs and the signals were recorded. The results are illustrated in Figs 3, S6, S7, and S8. As shown in Fig. 3, by increasing GSH concentration, the CL intensity of luminol-H_2_O_2_-QDs and luminol-H_2_O_2_-AgNPs increased linearly, while the CL intensity of luminol-AgNO_3_-(Cit-, BH_4_-) AuNPs decreased. As shown in Fig. S6, the results indicated that no significant changes in CL intensity of luminol-H_2_O_2_-AgNPs was observed by the addition of GSSG. It could be seen that GSSG has an inhibition effect on luminol-AgNO_3_-(Cit-, BH_4_-) AuNPs. Fig. S8(a1, b1, c1) clearly indicates that in luminol-H_2_O_2_-QDs CL system, the most intensive CL signal was obtained from GSH. Figure S7 shows that an opposite effect was observed for Cys: when the Cys concentration was lower than 35 μM, the CL intensity of luminol-H_2_O_2_- BH_4_-AuNPs increased with increasing Cys concentration; when Cys concentration was higher than 35 μM, the CL intensity decreased with increasing Cys concentration. A similar effect was observed for luminol-H_2_O_2_- Cit-AuNPs. Based on previous reports^41^,^44^,^45^,^46^,^47^,^48^ we suggested that the enhancement effect of low concentrations of Cys on luminol–AgNO_3_-AuNPs reaction may be due to the change of the gold nanoparticles surface-charge density. It is obvious that the negative charge density of AuNPs surface decreases after aggregation which occurs in the presence of Cys. On the other hand, luminol anion which is the molecular form of luminol in alkali media, can not easily interact with anionic AuNPs because of the electrostatic repulsion. Albeit it can more easily interact with aggregated AuNPs, causing a higher catalytic effect on the CL reaction. Thus, we reasoned that the enhanced catalysis of AuNPs occurring in low concentrations of Cys resulted from the decrease in the surface negative charge density of AuNPs. In contrast, in higher concentrations of Cys, an opposite effect was observed which is in agreement with previous reports stating that organic compounds such as Cys interact with gold nanoparticles in the CL reaction leading to an inhibition of CL intensity^2^,^13^,^49^. According to the proposed mechanism, reducing groups such as SH react with active oxygen intermediate radicals such as O_2_^**.−**^ and OH. The SH reducing groups actually compete with luminol for oxygen-containing intermediates, resulting in a decrease in CL intensity. Besides, the formation of Au–S covalent bonds, helps Cys strongly interacts with gold nanoparticles, decreasing the catalysis ability of nanoparticles for the formation of superoxide radical anion (O_2_^**.−**^) and luminol radicals (L^**.−**^) on the surface of gold nanoparticles. Consequently, a decrease in CL intensity occurs. The CL response curves of NPs-enhanced luminol CL upon the addition of GSH, GSSG, and Cys with the linear range for each of them are shown in Fig. S8.

### Application of NP-enhanced CL system for biothiol discrimination

The responses of various CL systems consisting four types of NPs and reagents to three different biothiols are shown in Fig. S8. The results revealed that the three biothoils with different molecular structures provided different binding capabilities toward nanoparticles, leading to a meaningful differences in the values of CL signals. CL signals are distinct, suggesting the possibility of biothiols discrimination using such a sensor array. The proposed sensor array was made of four sensor elements, and each of them shows different CL signal changes after interaction with biothiols. The ΔI patterns were subjected to linear discriminant analysis (LDA). These patterns were transformed to canonical scores which were visualized as a well-clustered two-dimensional plot as shown Fig. 4. We successfully discriminated biothiols solutions, for GSSG and GSH in the concentration range of 5.0–800.0 μM and for Cys in the concentration range of 25.0–100.0 μM by utilizing the training matrix generated from CL patterns. Three replicates were tested for each biothiol sample. They appeared exactly to their respective groups. Our system provided a sensitive way to differentiate biothiols at wide concentration ranges. The LDA method classified the biothiols into three distinct classes, revealing that nanoparticle-based chemiluminescence sensor array was capable of discriminating various biothiols.

By implementing hierarchical cluster analysis (HCA), a statistical classification method, the similarity of biothiols was analyzed. As shown in Fig. 5, the three biothiols appeared exactly to their respective groups.

Additionally, we achieved successful discrimination of individual thiols within their mixtures. Binary and ternary mixtures of thiols (at concentrations of 75.0 μM for each thiol) were tested. As shown in Fig. 6, LDA analysis on the mixtures of biothiols demonstrated that the canonical response patterns of the individual thiols and their mixtures were clearly clustered into seven groups.

### Fingerprints of biothiols generated via the CL sensor array based on various NPs

Fingerprints of Cys, GSH, and GSSG based on ΔI values were obtained from four nanoparticle coupled CL systems of luminol–H_2_O_2_-CdTe QDs, luminol–H_2_O_2_- Cit-AgNPs, luminol–AgNO_3_- Cit-AuNPs, and luminol–AgNO_3_- BH_4_-AuNPs. ΔI = I − I_0_, where I and I_0_ refer to the value of CL intensity in the presence and absence of biothiols, respectively. As shown in Fig. 7, the results confirmed that different biothiols affect CL responses with different degrees, leading to characteristic response patterns. We also employed the heat maps to visualize the ability of that this sensor in generating characteristic fingerprints for each of the three biothiols. A heat map is a 2D representation of data in which individual values in a matrix are represented as colors. As a visual tool, heat maps can be very powerful because it provides an immediate visual summary of information of data values that would be much harder to understand if presented numerically. The color key (right side of the Fig. 8) is a continuous range. In the color scale, a dark blue is for the lowest value and a dark red is for the highest value. A color range moves from blue to red as the values increase, giving the appearance of getting hotter. The heat map depicted in Fig. 8 visualizes the values of ΔI which were obtained from the four sensor elements for Cys, GSH, and GSSG at the concentrations of 50.0 μM larger values are represented by dark red squares while smaller values are shown by darker blue ones. With this system of color-coding to represent the values, one can easily make quick comparisons between the three analytes and distinguish CL responses patterns of each of them.

### Blood plasma analysis

In order to verify the applicability of the developed method, the proposed procedure was applied for the analysis of analytes in real sample. Human plasma sample was spiked with different concentrations of analytes. As shown in Fig. 9, LDA analysis demonstrated that the proposed array could successfully discriminate between cysteine, glutathione and glutathione disulfide in the concentration range of 5.0–800.0 μM of GSH, 15.0–800.0 μM of GSSG, and 25.0–100.0 μM of Cys in human plasma.

## Conclusions

In summary, we have designed a chemiluminescence sensor array on the basis of distinct CL responses patterns generated by different nanoparticles in order to discriminate biothiols for the first time. The CL sensor array can successfully discriminate among three selected biothiols in a wide concentration range. The developed sensor array based on various NPs as sensor elements, opens a new window to the use of nanoparticle-based CL systems constructing various sensing arrays. We believe by varying the CL reagents or NPs as sensor elements, the nanoparticle-based chemiluminescence sensor array can emerge new opportunities for various biomolecules discrimination.

## Methods

### Reagents

Hydrogen tetrachloroaurate (HAuCl_4_.3 H_2_O (99.5%)), thioglycolic acid (TGA, 99%), cadmium chloride (CdCl_2_. 2H_2_O), and tellurium powder (Te, 99.8%) were purchased from Sigma. Sodium borohydride (NaBH_4_, 98%), silver nitrate (AgNO_3_), sodium hydroxide (NaOH), hydrochloric acid (HCl), sodium citrate (99%), glutathione (GSH, 98%), glutathione disulfide (GSSG, 98%), cysteine (Cys, 97%), hydrogen peroxide (H_2_O_2_, 30%), and 5-amino-1,2,3,4-tetrahydro-1,4-phthalazinedione (Luminol, 95%) were purchased from Merck. Human blood plasma samples were obtained from the Tehran Blood Transfusion Service (Tehran, Iran). Milli-Q grade water (18.2 MΩ.cm at 25 °C) was used for the preparation of all aqueous solutions.

### Instrumentation

UV-Vis spectra were measured and recorded with a Lambda (Perkin Elmer, USA) spectrophotometer with the use of 1.0 cm cell. The transmission electron microscopy (TEM) images were taken using a (PHILIPS MC 10 TH microscope at an acceleration voltage of 100 kV). Size distributions of the particles were measured using Zetasizer Viscotec 802 at ambient temperature.

### Synthesis of different NPs

#### Synthesis of citrate-coated AuNPs

Cit-AuNPs with a concentration of 8.16 nM were prepared according to Turkevich method^50^. In regard to the modified Turkevich method, after bringing 50.0 mL of HAuCl_4_ solution (1.0 mM) to boil, 5.0 mL of sodium citrate (38.8 mM) was added. The mixture was refluxed for 30 min. Then ice-cold water was quickly used for cooling it and finally stored at 4 °C temperature. The synthesized AuNPs were characterized by TEM, DLS, and UV–vis spectroscopy, for which the results are shown in Figs S9a, S10a and S11a.

#### Synthesis of NaBH_4_ coated AuNPs

BH_4_ –AuNPs with a concentration of 1524 nM were prepared as follow^51^. Briefly, 100.0 μL 50.0 mM mixture solution of HAuCl_4_ and HCl was added, with vigorous stirring, to 9.6 mL DI water. Then, 400.0 μL 50.0 mM mixture solution of NaBH_4_ and NaOH was added and stirred for 15 min. The synthesized AuNPs were characterized by TEM, DLS, and UV–vis spectroscopy, and the results are shown in Figs S9b, S10b and S11b.

#### Synthesis of citrate-capped AgNPs

Cit-AgNPs with a concentration of 1.67 nM were synthesized by the reduction of AgNO_3_ solution using citrate according to a previous report^52^. Typically, 250.0 μL 100.0 mM AgNO_3_ solution was added to 100.0 mL DI water. Then, 250.0 μL 100.0 mM sodium citrate solution and then 1.0 mL 5.0 mM sodium borohydride solution were respectively added to the solution and it was stirred for 30 min. The resulting mixture was stored in the dark for 24 h before use. The synthesized AgNPs were characterized by DLS, and UV–vis spectroscopy, and the results are shown in Figs S10d and S11c.

#### Synthesis of TGA-capped CdTe QDs

In brief, 0.256 g of CdCl_2_·5 H_2_O and 200.0 μL TGA were dissolved in 4.0 mL of ultrapure water. The pH was adjusted to 9.0 with 3.0 M NaOH. Meanwhile, 0.065 g of Te powder and 0.183 g of NaBH_4_ were added into 75.0 mL of ultrapure water under Ar flow. The solution was heated (∼150 °C) and refluxed. Heating of the solution under reflux was continued until the boiling solution’s color changed to purple. Then the Cd solution was added to the purple solution. TGA-CdTe QDs solution with yellow fluorescence and with a concentration of 47.52 μM was prepared after 4 h^53^. The synthesized QDs were characterized by DLS, and the result is shown in Fig. S10c.

#### Procedure of detection

**Figure 1:**
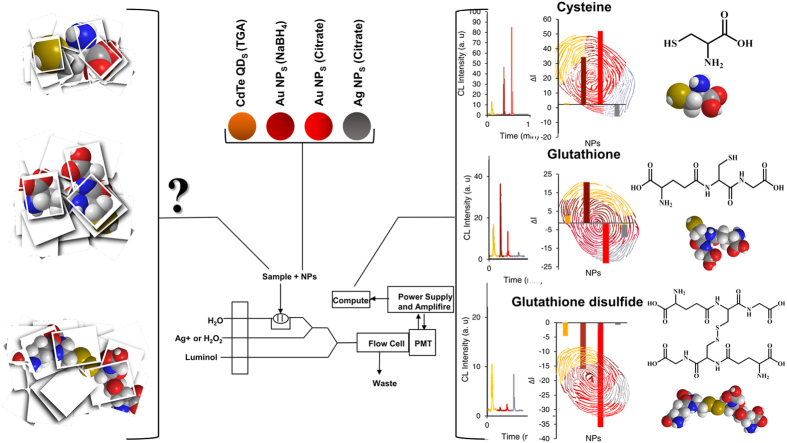
Structure of the system platform. Chemical structures of Cys, GSH and GSSG and flow injection CL system, generating distinct response patterns for each analyte.

**Figure 2:**
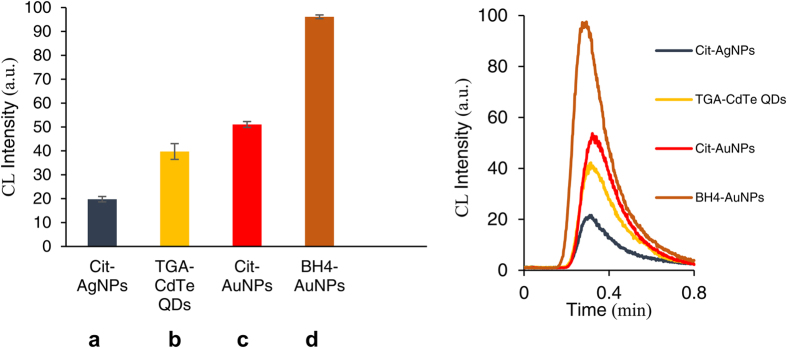
Enhancement effect of the nanoparticles on the CL systems. (**a,b**) The luminol–H_2_O_2_ CL system (0.3 mM luminol, 0.15 M H_2_O_2_, 20 rpm flow rate, NaOH concentration of luminol solution = 0.01 M). (**c,d**) The luminol– AgNO_3_ CL system (0.5 mM luminol, 25.0 μM AgNO_3_, 20 rpm flow rate, NaOH concentration of luminol solution = 0.1 M).

**Figure 3:**
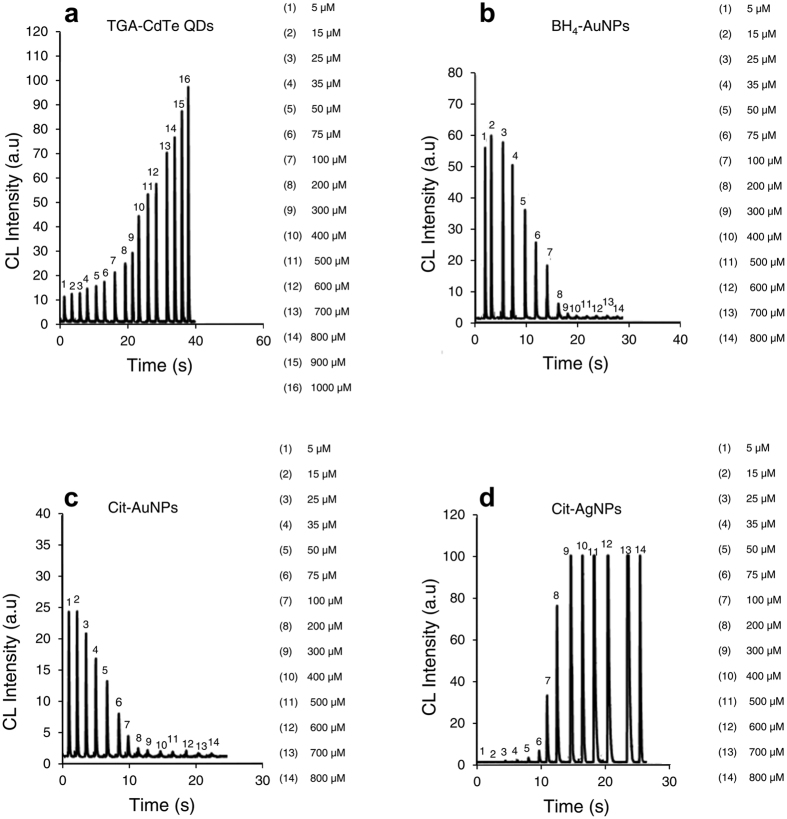
The CL signals of different nanoparticle enhanced CL systems after adding different concentrations of GSH. (**a**) TGA-CdTe QDs, (**b**) BH_4_-AuNPs, (**c**) Cit-AuNPs, and (**d**) Cit-AgNPs enhanced luminol CL systems. Numbers 1, 2, 3, … show the GSH concentrations.

**Figure 4:**
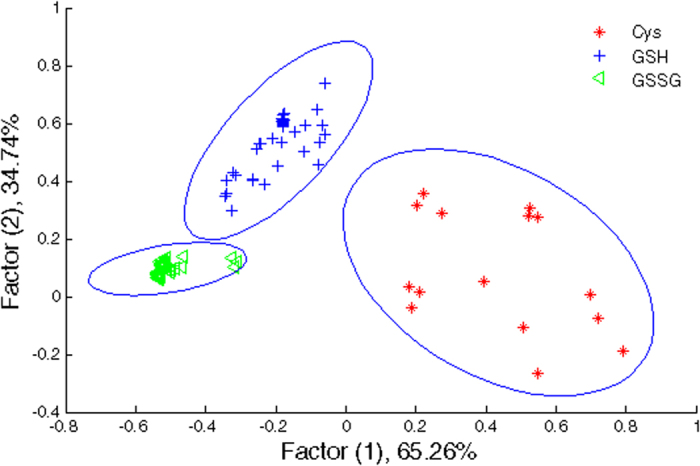
LDA plot for the discrimination of GSSG, GSH, and Cys. The concentration ranges are 5.0–800.0 μM for GSH, 5.0–800.0 μM for GSSG, and 25.0–100.0 μM for Cys.

**Figure 5:**
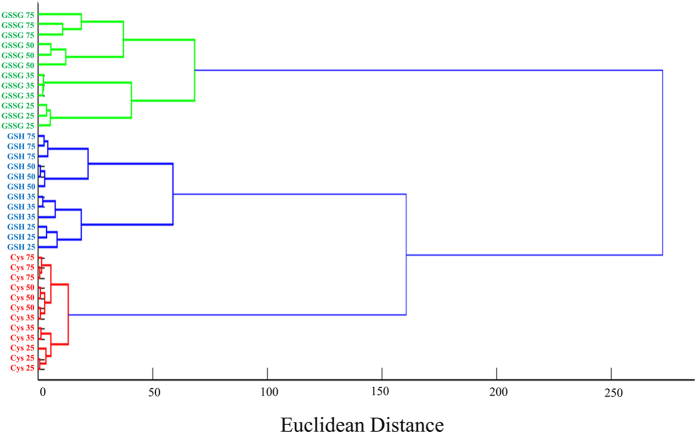
HCA dendrogram for biothiols. Cys, GSH, and GSSG at four concentration levels with three replicates.

**Figure 6:**
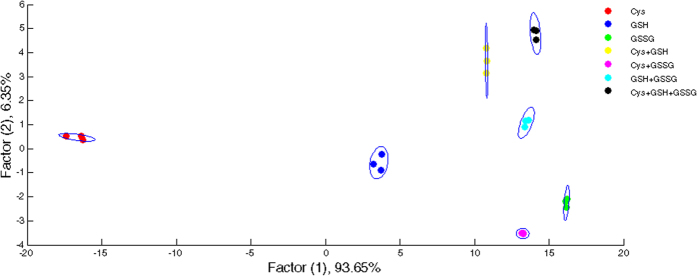
Two-dimensional score plot for the discrimination of GSSG, GSH, and Cys and their mixtures. Discrimination of individual thiols and their binary and ternary mixtures. Thiol concentrations are 75.0 μM.

**Figure 7:**
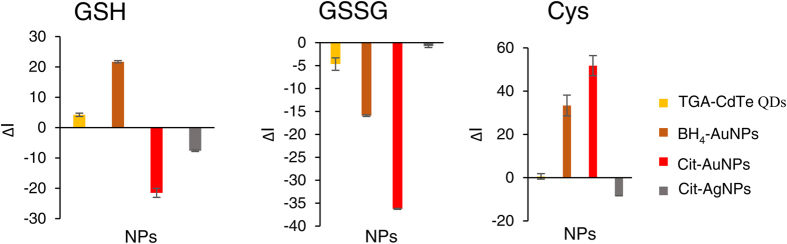
Varying CL responses in the presence of biothiols. 50.0 μM of Cys, GSH, and GSSG is added.

**Figure 8:**
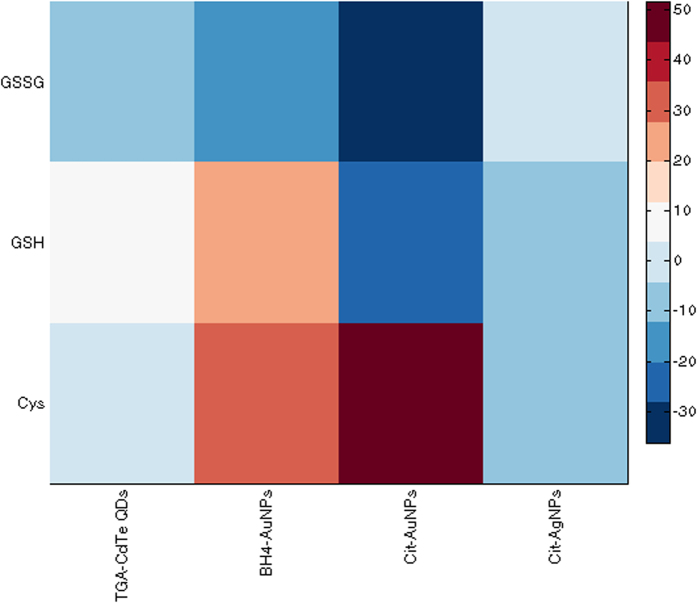
Heat map generated from the CL response of different biothiols. 50.0 μM of Cys, GSH, and GSSG is added.

**Figure 9:**
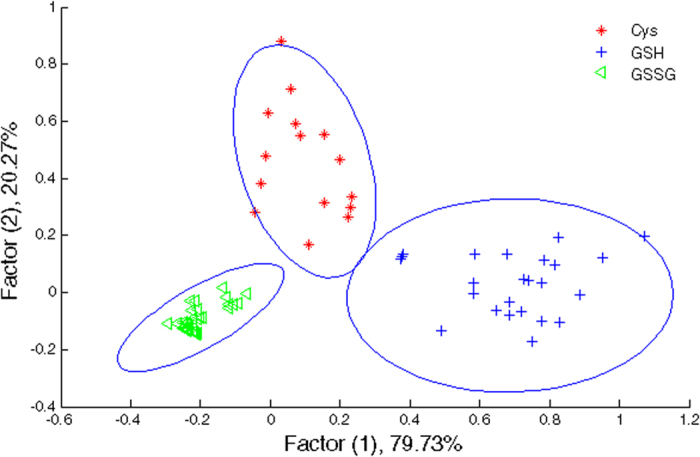
Two-dimensional LDA plot for the discrimination of GSSG, GSH, and Cys in human plasma. The concentration ranges include 5.0–800.0 μM of GSH, 15.0–800.0 μM of GSSG, and 25.0–100.0 μM of Cys.

The diagram shown in Fig. 1 illustrates the laboratory-built flow injection CL system employed in this work. The NPs solution was injected into the ultrapure water as a carrier stream through a 100.0 μL loop-valve injector, mixed with luminol, and H_2_O_2_ (or AgNO_3_) solutions through three-way pieces. Then, the mixed solution moved to a spiral-shaped flow cell which was located in front of a photomultiplier tube (PMT). The PMT signal was imported to the computer and the CL signal was monitored.

#### Fabrication of array-based sensing system for biothiols discrimination

For discrimination of biothiols, three biothiols with concentrations ranging from 5.0 to 800.0 μM were tested against the four NPs. Three replicates were tested for each of them. The variations in CL intensity (ΔI = I − I_0_) were used to generate the response pattern, where I and I_0_ are in order the value of CL signal in the presence and absence of the biothiols.

The stock solutions of three biothiols were prepared in DI water. For each biothiol, AgNPs solution (50.0 μL) was added to 100.0 μL biothiol solution and TGA-CdTe QDs solution (30.0 μL) was added to 90.0 μL biothiol solution. Either AgNPs or QDs-biothiols solutions were injected into the carrier stream (ultrapure water) through a 100.0 μL loop-valve injector, mixed with H_2_O_2_ and luminol solutions through three-way pieces. Then the mixed solution flowed via a spiral-shaped flow cell positioned in front of a photomultiplier tube. In addition, the BH_4_-AuNPs solution (20.0 μL) was added to biothiol solution (140.0 μL) and Cit-AuNPs solution (50.0 μL) was added to biothiol solution (100.0 μL). The AuNPs-biothiols solutions were injected into the carrier stream (ultrapure water) through a 100.0 μL loop-valve injector, mixed with AgNO_3_ and luminol solutions through three-way pieces.

#### Preparation of human plasma samples

Trichloroacetic acid (TCA) was added to the freshly thawed human plasma sample and the mixture was stirred vigorously to precipitate proteins. After centrifuging at ~10,000 rpm for 10 min at room temperature, the clear supernatant was diluted 20 times and the subsequent was subjected to assay under the optimized conditions.

## Additional Information

**How to cite this article**: Shahrajabian, M. and Hormozi-Nezhad, M. R. Design a New Strategy Based on Nanoparticle-Enhanced Chemiluminescence Sensor Array for Biothiols Discrimination. *Sci. Rep.* **6**, 32160; doi: 10.1038/srep32160 (2016).

## Supplementary Material

Supplementary Information
