# Supplementary material for: Design a New Strategy Based on Nanoparticle-Enhanced Chemiluminescence Sensor Array for Biothiols Discrimination
Source: Sci Rep. 2016 Aug 30;6:32160. doi: 10.1038/srep32160 (PMC5004156; doi:10.1038/srep32160)
Supplement: Supplementary Information [file srep32160-s1.doc]

**Design a New Strategy Based on Nanoparticle-Enhanced Chemiluminescence Sensor Array for Biothiols Discrimination**

**Maryam Shahrajabian^1^, M. Reza Hormozi-Nezhad^1, 2*^**

^1^Department of Chemistry, Sharif University of Technology, Tehran, 11155-9516, Iran

^2^Institute for Nanoscience and Nanotechnology, Sharif University of Technology, Tehran, Iran

*Email: [hormozi@sharif.edu](mailto:hormozi@sharif.edu)


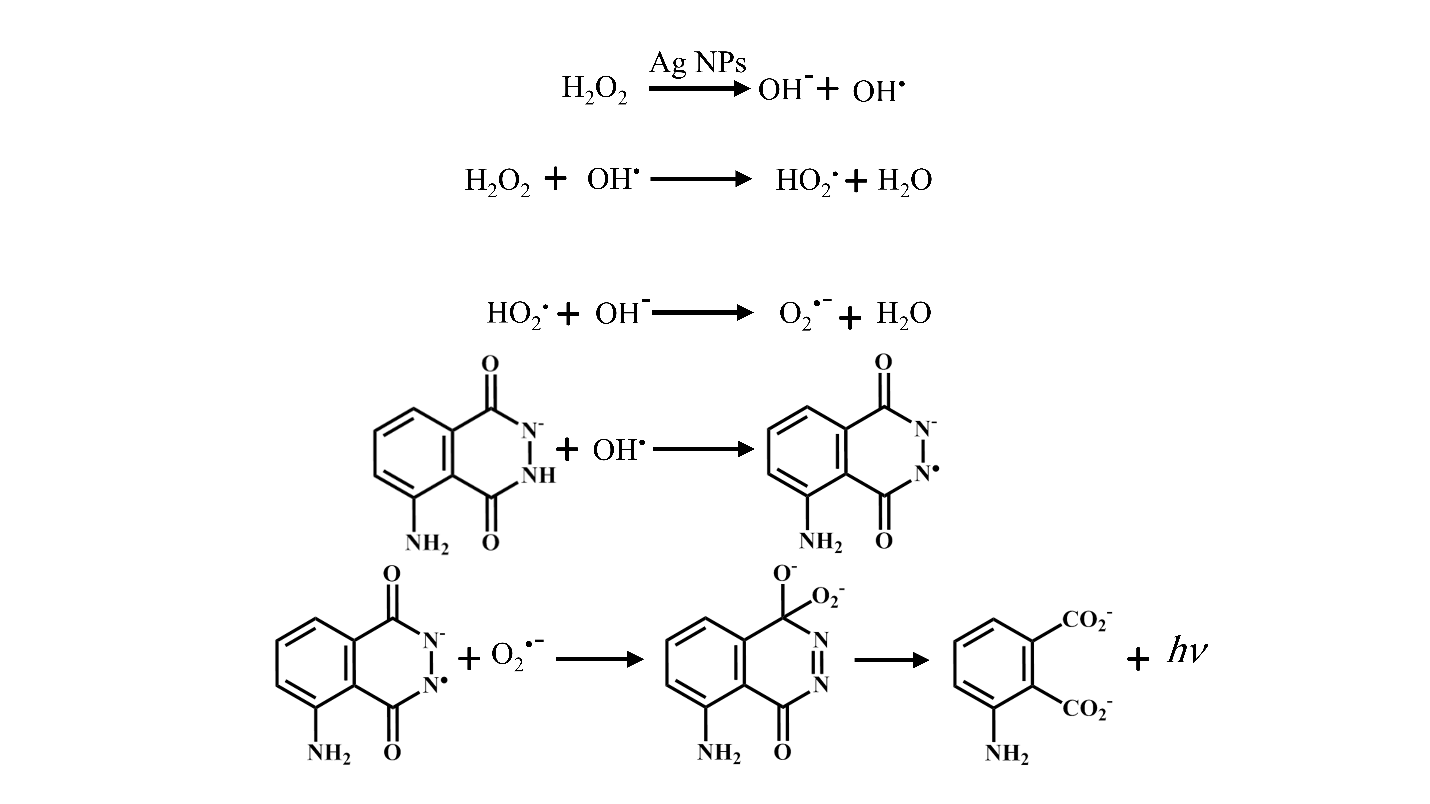


**Figure S1. Possible mechanism for the luminol–H_2_O_2_–AgNPs CL system.**


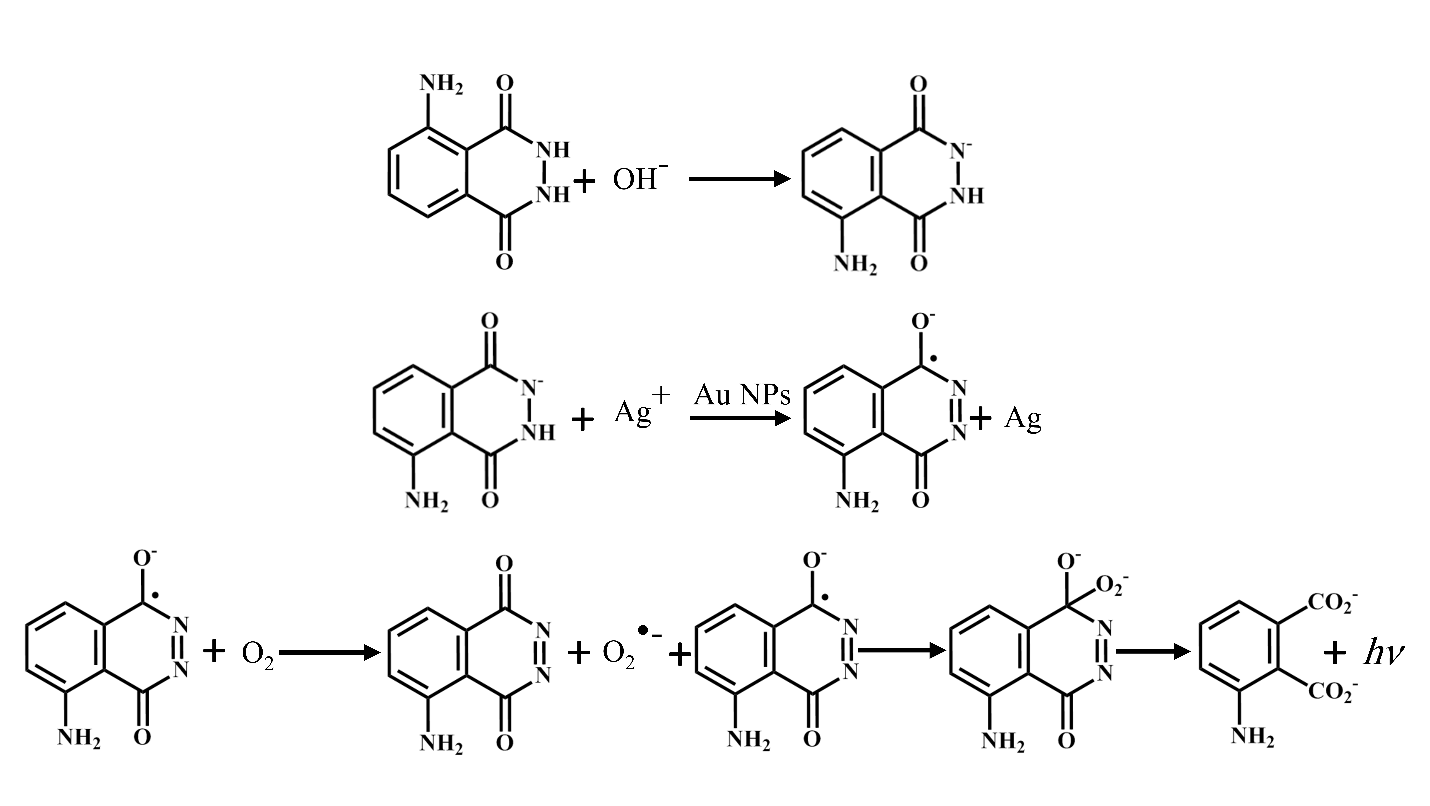


**Figure S2. Possible mechanism for the luminol–AgNO_3_–AuNPs CL system.**


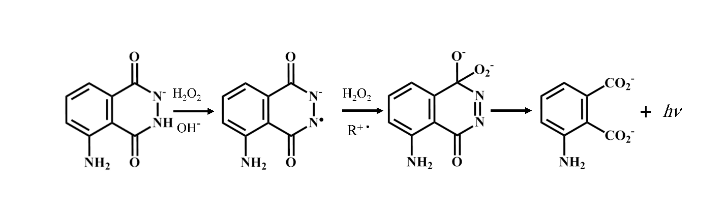


**Figure S3. Possible luminol CL mechanism in the presence of CdTe QDs and H_2_O_2_.** (R^+•^ refers to cation radicals (CdTe QDs)^+•^).


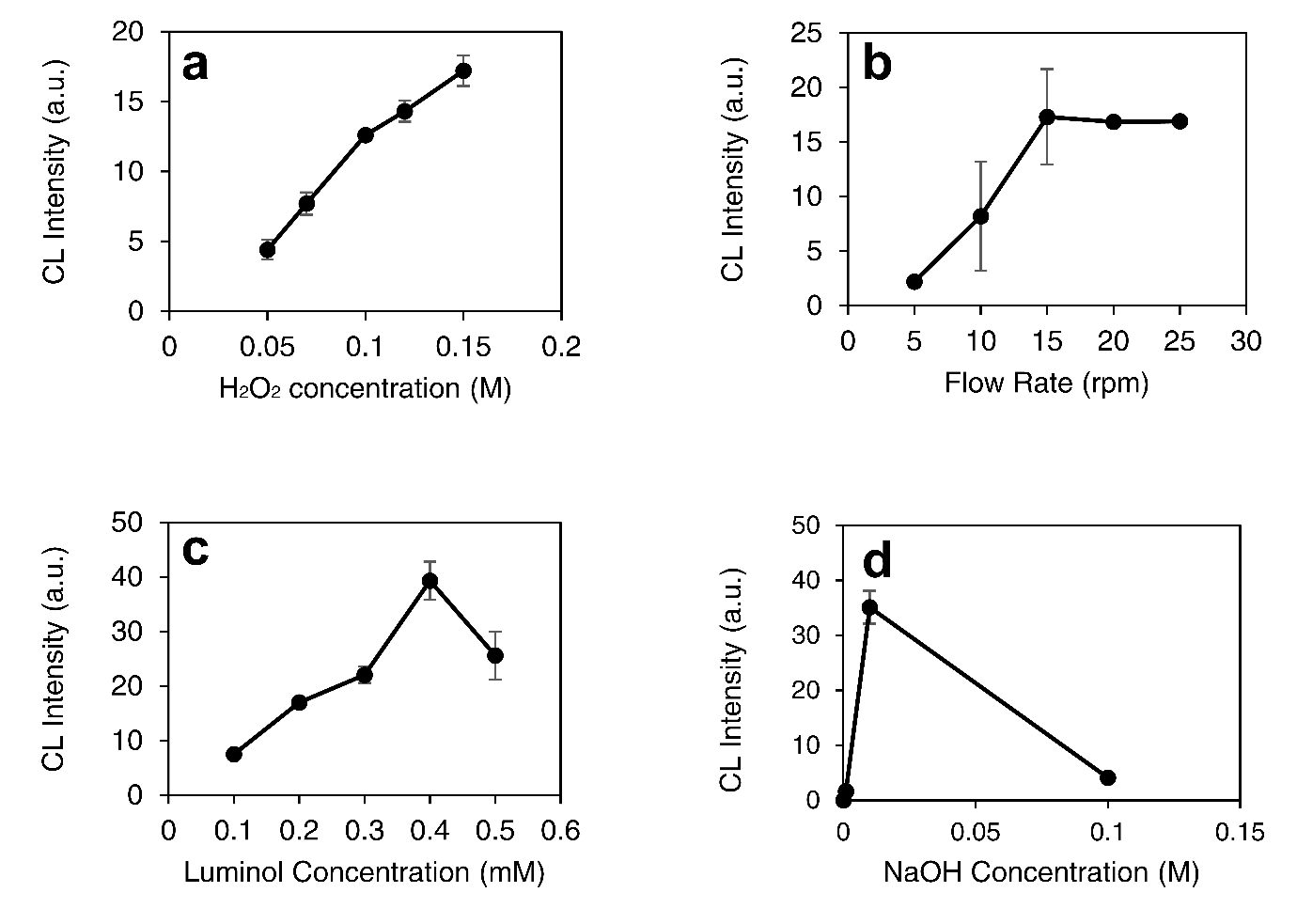


**Figure S4. Effects of the experimental conditions on the luminol–H_2_O_2_–AgNPs CL system.** (**a**) Effect of H_2_O_2_ concentration, 0.2 mM luminol, 20 rpm flow rate, 0.01 M NaOH. (**b**) Effect of flow rate, 0.2 mM luminol, 0.15 M H_2_O_2_, 0.01 M NaOH. (**c**) Effect of luminol concentration, 0.15 M H_2_O_2_, 20 rpm flow rate, 0.01 M NaOH. (**d**) Effect of NaOH concentration of luminol solution, 0.4 mM luminol, 0.15 M H_2_O_2_, 20 rpm flow rate, 0.01 M NaOH.

**
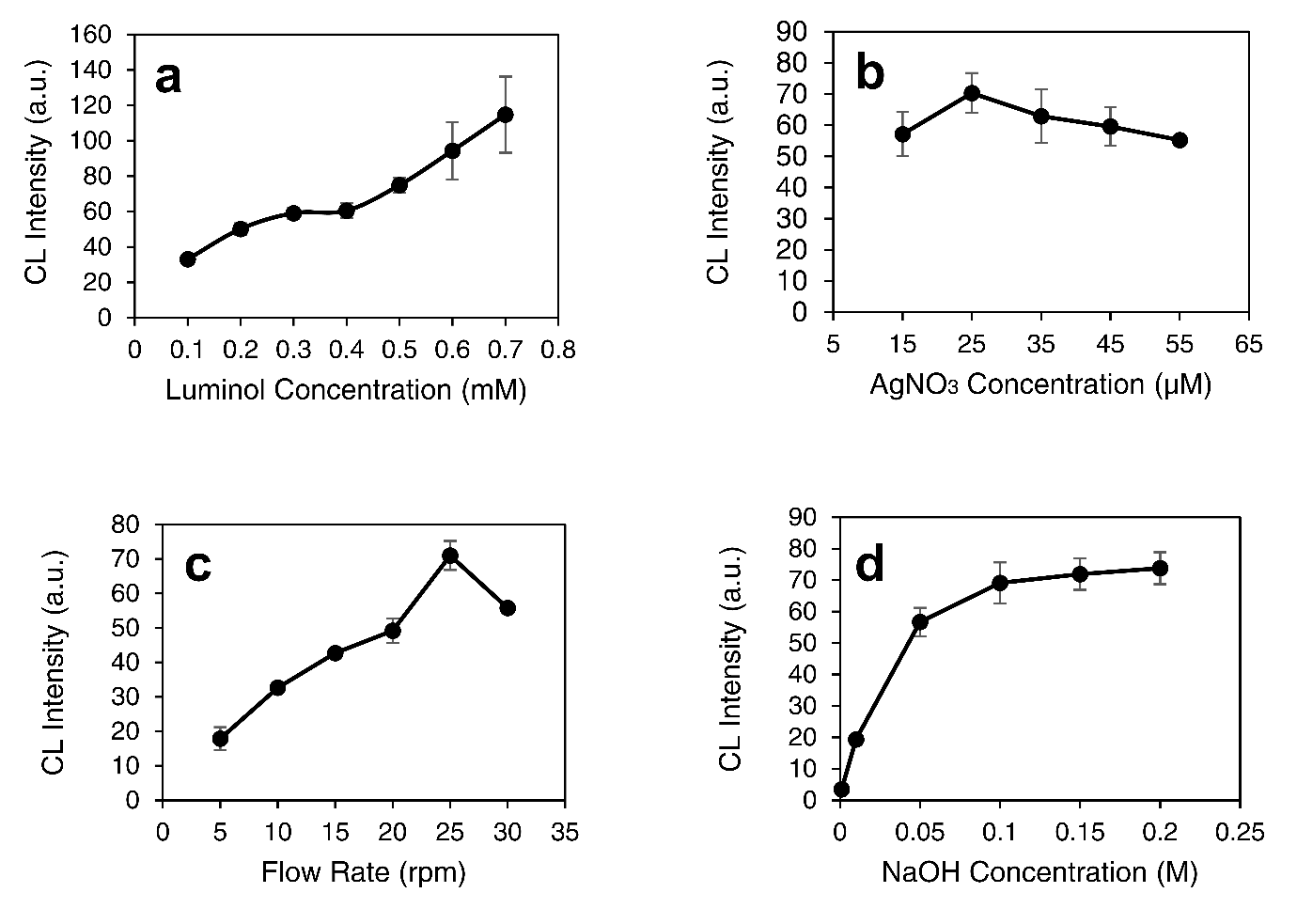
**

**Figure S5. Effects of the experimental conditions on the luminol–AgNO_3_– Cit-AuNPs CL system.** (**a**) Effect of luminol concentration, 25.0 µM AgNO_3_, 25 rpm flow rate, 0.1 M NaOH. (**b**) Effect of AgNO_3_ concentration, 0.5 mM luminol, 25 rpm flow rate, 0.1 M NaOH. (**c**) Effect of flow rate, 0.5 mM luminol, 25 µM AgNO_3_, 0.1 M NaOH. (**d**) Effect of NaOH concentration of luminol solution, 0.5 mM luminol, 25 µM AgNO_3_, 25 rpm flow rate.


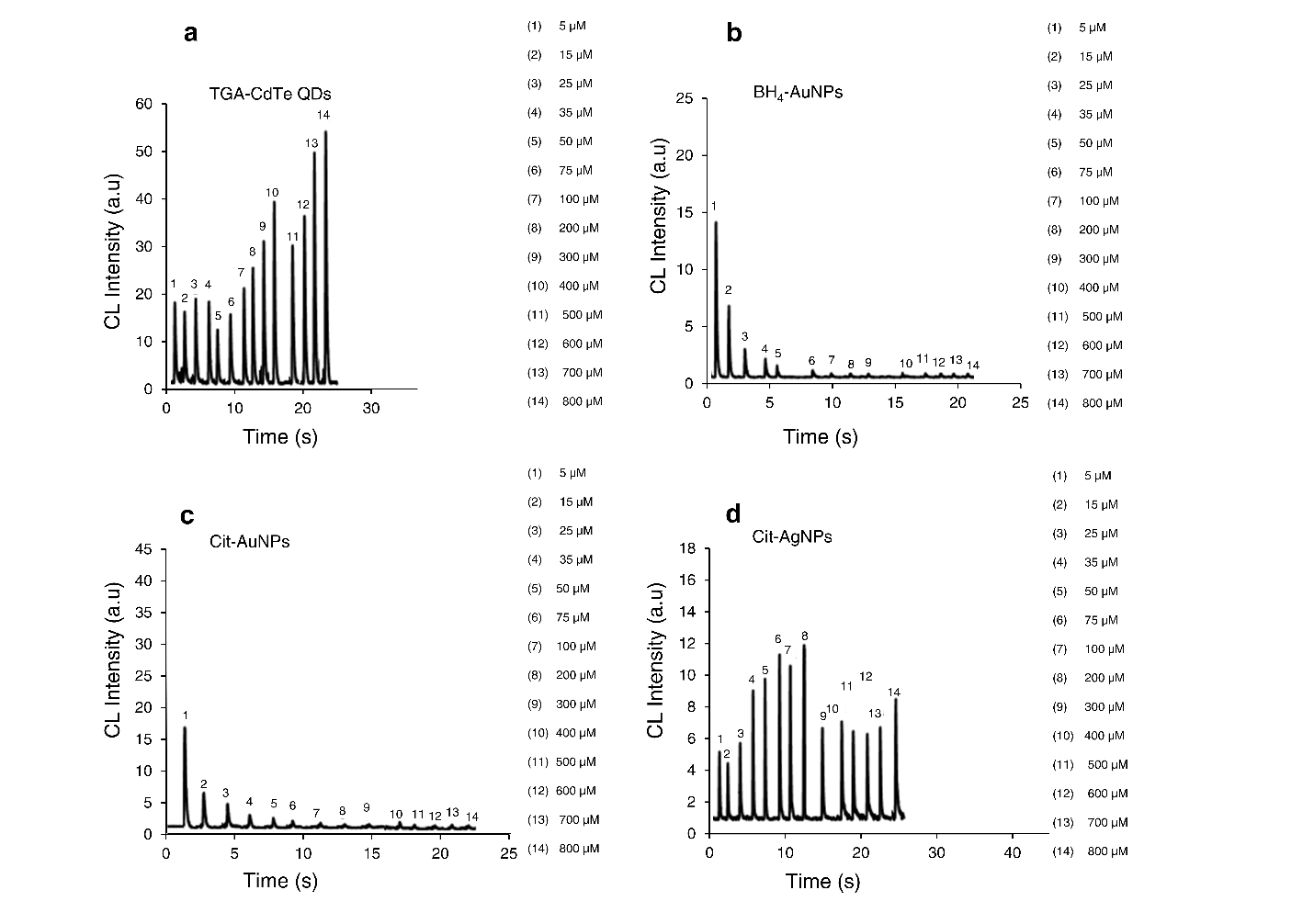


**Figure S6. The CL signals of different nanoparticle enhanced CL systems after adding different concentrations of GSSG.** (**a**) TGA-CdTe QDs, (**b**) BH_4-_AuNPs, (**c**) Cit-AuNPs, and (**d**) Cit-AgNPs enhanced luminol CL systems. Numbers 1, 2, 3,… show the GSSG concentrations.


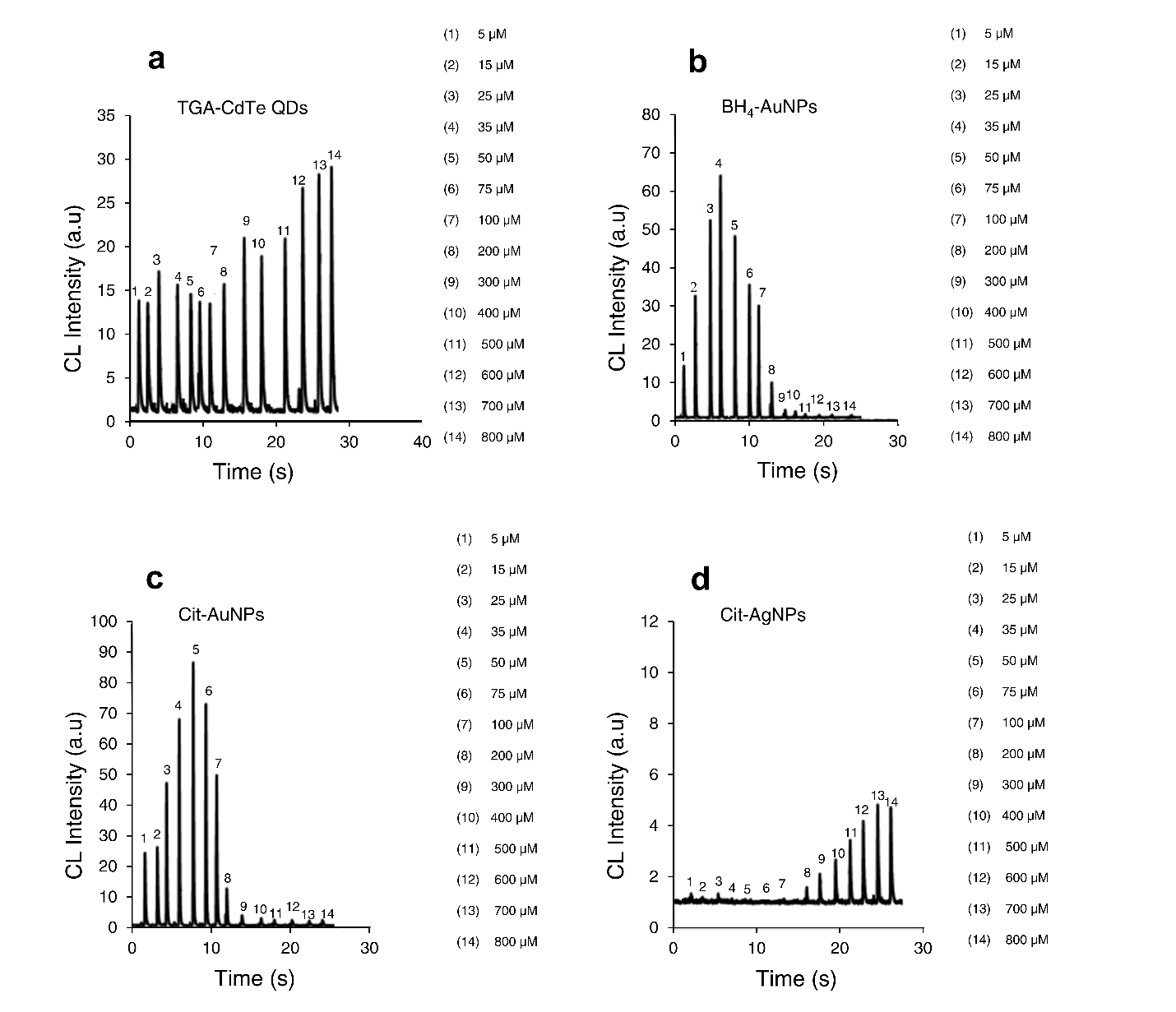


**Figure S7. The CL signals of different nanoparticle enhanced CL systems after adding different concentrations of Cys.** (**a**) TGA-CdTe QDs, (**b**) BH_4_-AuNPs, (**c**) Cit-AuNPs, and (**d**) Cit-AgNPs enhanced luminol CL systems. Numbers 1, 2, 3,… show the Cys concentrations.


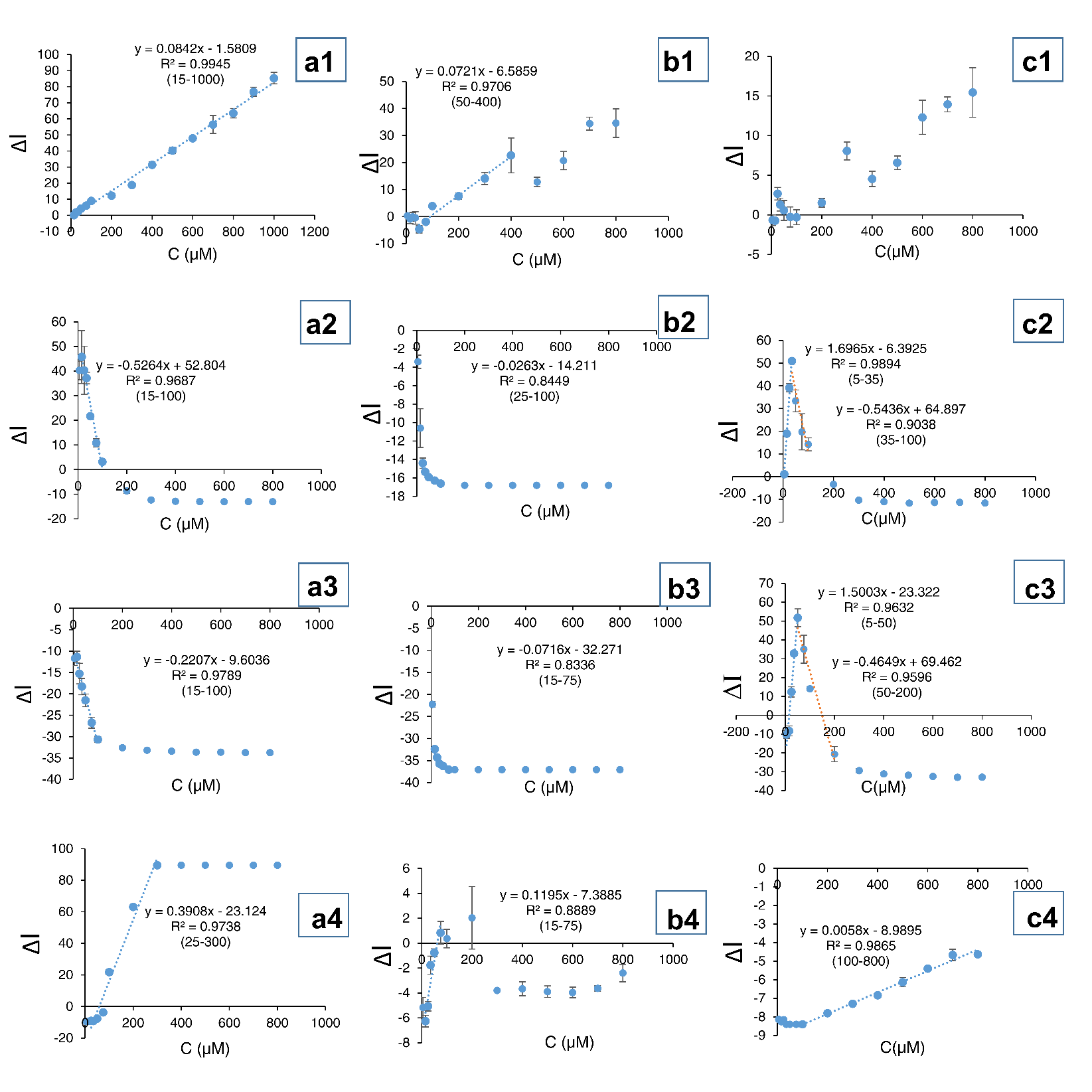
**FigureS8. The CL response curves of the analytes.** Addition of (**a**) GSH, (**b**) GSSG, and (**c**) Cys in different concentrations (5.0-800.0 µM), to (**1**) TGA-CdTe QDs, (**2**) BH_4_-AuNPs, (**3**) Cit-AuNPs, and (**4**) Cit-AgNPs-enhanced luminol CL systems. Linear dynamic ranges are written in parentheses. (Concentration range is 5.0-1,000 µM for GSH in luminol-H_2_O_2_- CdTe QDs CL system).


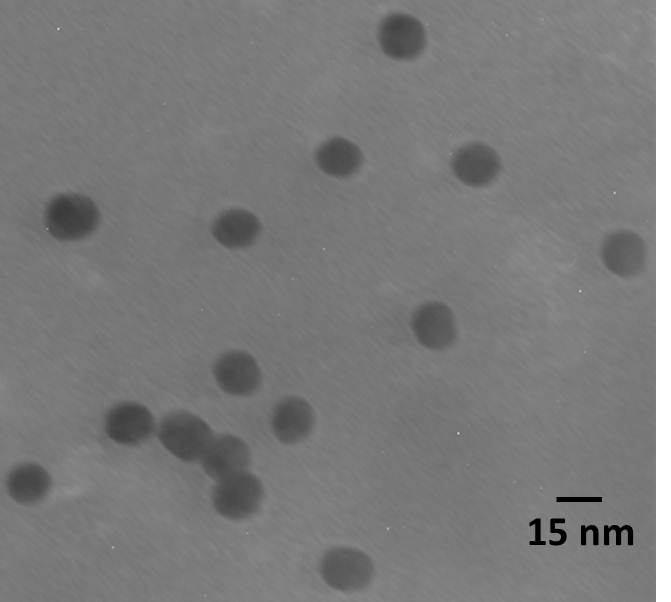

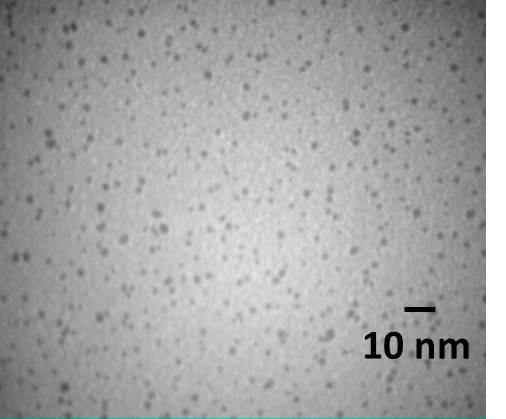


**(a)**

**(b)**

**Figure S9. TEM images of nanoparticles.** (**a**) Cit-AuNPs, and (**b**) BH_4_-AuNPs.


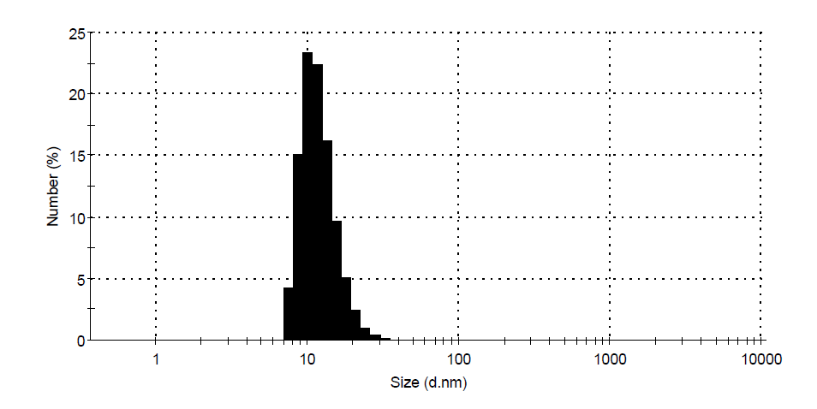


**(a)**


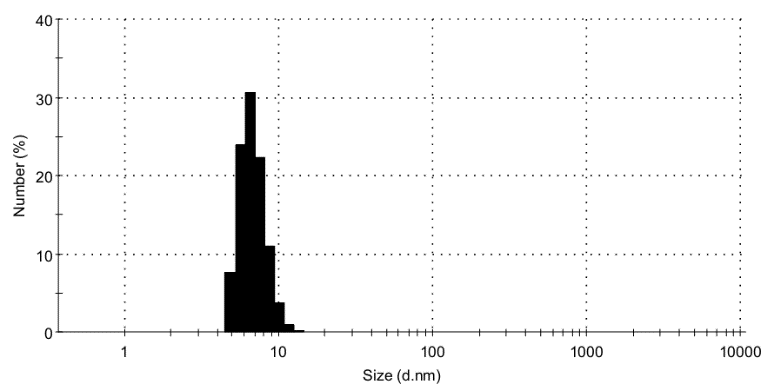


**(b)**


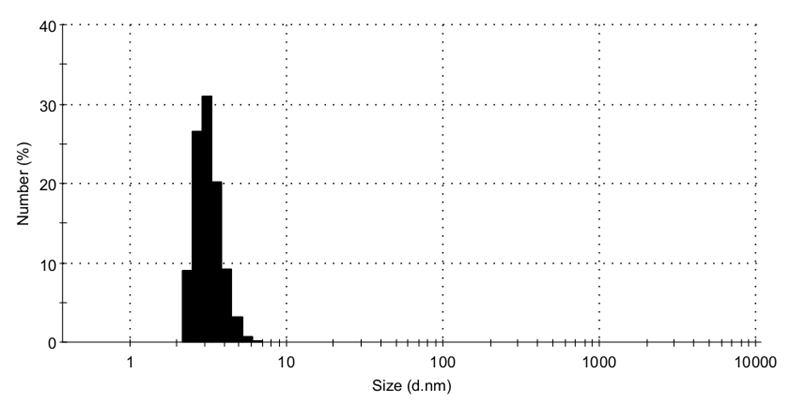


**(c)**


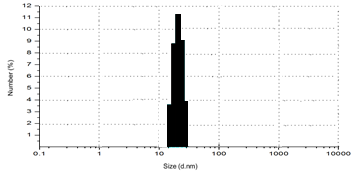


**(d)**

**Figure S10.**  **Number size distribution of the nanoparticles**. (**a**) Cit- AuNPs, (**b**) BH_4_-AuNPs, (**c**) TGA-CdTe QDs, and (**d**) Cit-AgNPs.


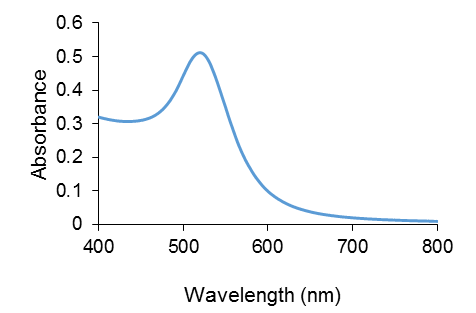


**(a)**

**
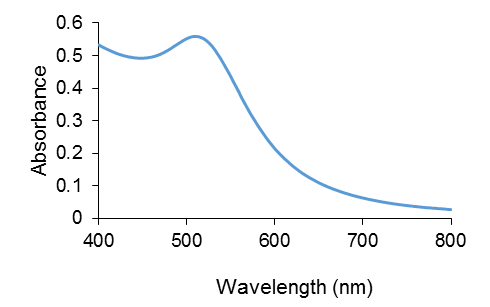
**

**(b)**

**
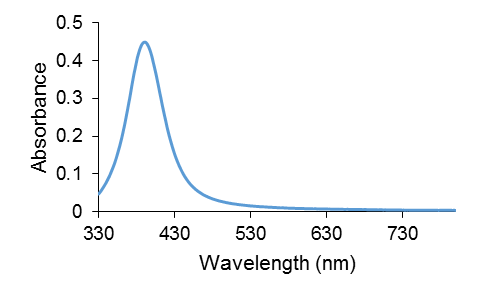
**

**(c)**

**Figure S11. UV-Vis spectra of the nanoparticles.** (**a**) Cit-AuNPs, (**b**) BH_4_-AuNPs, and (**c**) Cit-AgNPs.
